# A Narrative Review on the Pathophysiology and Management for Radiation Cystitis

**DOI:** 10.1155/2015/346812

**Published:** 2015-12-22

**Authors:** C. Browne, N. F. Davis, E. Mac Craith, G. M. Lennon, D. W. Mulvin, D. M. Quinlan, Gerard P. Mc Vey, D. J. Galvin

**Affiliations:** ^1^Department of Urology, St. Vincent's University Hospital, Dublin 4, Ireland; ^2^Department of Radiation Oncology, St. Vincent's University Hospital, Dublin 4, Ireland

## Abstract

Radiation cystitis is a recognised complication of pelvic radiotherapy. Incidence of radiation cystitis ranges from 23 to 80% and the incidence of severe haematuria ranges from 5 to 8%. High quality data on management strategies for radiation cystitis is sparse. Treatment modalities are subclassified into systemic therapies, intravesical therapies, and hyperbaric oxygen and interventional procedures. Short-term cure rates range from 76 to 95% for hyperbaric oxygen therapy and interventional procedures. Adverse effects of these treatment strategies are acceptable. Ultimately, most patients require multimodal treatment for curative purposes. Large randomised trials exploring emergent management strategies are required in order to strengthen evidence-based treatment strategies. Urologists encounter radiation cystitis commonly and should be familiar with diagnostic modalities and treatment strategies.

## 1. Introduction

Radiotherapy is a common treatment modality for the management of pelvic malignancies. Treatment for prostate, bladder, rectal, and cervical cancers often involves radiotherapy and radiation cystitis is a recognised complication. The response of the urinary bladder to radiation treatment can be classified into acute or subacute reactions that occur within three to six months of radiation treatment and late reactions that occur after six months. Urinary symptoms include dysuria, frequency, urgency, and haematuria [1]. Haemorrhagic cystitis is a recognised late complication of pelvic radiation and is commonly encountered by urologists. Modern technologies in radiotherapy aim to reduce the dose administered to organs at risk, including the bladder. Differences in toxicities between radiotherapy modalities are explained by total radiation dose to the genitourinary tract [2]. The bladder is sensitive to small increments of radiation dose [3]. The introduction of intensity modulated radiotherapy (IMRT) has allowed reduction in planning target volume (PTV) margins, resulting in significantly less genitourinary toxicity compared to 3D conformal radiotherapy [4–7]. Stereotactic radiotherapy has acceptable and comparable toxicity rates to external beam radiotherapy [8]. In this narrative review, we discuss the pathophysiology, epidemiology, and management options for radiation cystitis. Our aim is to objectively assess the systemic, intravesical, and surgical management options for this problematic condition.

## 2. Materials and Methods

A literature search was performed using PubMed database and the Cochrane Central Register of Controlled Trials, aiming to identify peer-reviewed articles that outlined epidemiology, pathophysiology, diagnosis, and management of radiation cystitis. The following terms were entered into the search function for each database: “radiation” AND “cystitis” OR “radiation cystitis”. One hundred and fifty-four studies published between 1953 and 2015 were identified. The bibliographies of these studies were interrogated to identify other studies for possible inclusion.

The latest search was conducted on 24 September 2015. One author (CB) examined the title and abstract of all retrieved studies and obtained full texts of all papers for potential inclusion. These full texts were analysed for suitability for reporting. American Urological Association and European Association of Urology guidelines were also included. Case reports were excluded. A total of fifty-five papers were included for review.

## 3. Epidemiology

The reported incidence of radiation cystitis varies from 23% to 80% and this wide range is due to variable types and doses of radiotherapy among different medical subspecialties [1]. The mean duration for developing radiation cystitis is 31.8 months after treatment [10]. Males are more likely to develop radiation cystitis than females (2.8 : 1) due to frequent use of radiotherapy in the treatment of prostate cancer [10]. The reported incidence of severe haematuria ranges from 5% to 8% and haematuria can develop up to fourteen years following radiotherapy treatment [9, 28–30]. The actuarial risk of developing haematuria is 5.8% at five years and 9.6% at twenty years [31].

## 4. Pathophysiology

The pathophysiology of radiation cystitis is poorly understood. Multiple mechanisms of radiation damage to the bladder have been described (Table 1). The proposed mechanisms of pathology include inflammatory effects of ionising radiation traumatising the urothelium, vasculature, and detrusor muscle [10] with radiation effects on the urothelium most evident after four months [1].

Postradiation intermediate and basal urothelium displays nuclear irregularity and cellular oedema. The tight junctions and polysaccharide layer are disrupted, allowing hypertonic urine and isotonic tissue in contact with one another which results in tissue inflammation and early symptomatic acute radiation cystitis [1]. Vascular endothelial cells are thought to be the main target cell for bladder damage after radiation with late bladder fibrosis occurring secondary to vascular ischaemia of the bladder wall [1]. Vascular endothelial cell oedema is evident at three months and endothelial cell proliferation is evident after six months. Vascular ischaemia, oedema, and cellular destruction cause replacement of bladder smooth muscle fibres with fibroblasts and lead to increased collagen deposition and subsequent decreases in bladder compliance and capacity [9].

## 5. Diagnosis

The diagnosis of radiation cystitis is mainly based on exclusion of other causes of the patient's symptoms. An initial assessment involves a full patient history, physical examination, urinalysis, urine culture, urine cytology, and cystoscopy. Computed tomography (CT) may exclude an upper tract lesion as the cause of haematuria and magnetic resonance imaging (MRI) should be considered in the presence of a previous pelvic malignancy [32]. The Radiation Therapy Oncology Group guidelines on late radiation morbidity outline a grading system for radiation cystitis [11] (Table 2).

## 6. Treatment

A number of treatment options are described for radiation cystitis. However, a multitude of these are anecdotal and presented in case reports or very small case series and the paucity of high quality evidence in the form of randomised control trials makes development of meaningful evidence-based treatment algorithms difficult (Table 5). Management options can be divided into systemic treatments, intravesical treatments, and interventional procedures.

### 6.1. Systemic Treatments

As outlined above, one of the proposed mechanisms for the pathophysiology of radiation cystitis is disruption of the urothelium which affects the integrity of the bladder-urine interface and propagates inflammation that causes bladder damage. The rationale behind systemic treatments is to replace or enhance the polysaccharide layer of the bladder and reduce vascular fragility. Glucosamine and pentosan polysulfate have been postulated as possible systemic treatment options, but there is no evidence in the literature to support this [31]. Outlined below are some of the more commonly used agents and their relevant evidence (Table 3).

#### 6.1.1. TCDO/WF10

TCDO/WF10 is a chemically stabilised chlorite matrix that has previously been shown to have a positive effect in chronic inflammatory conditions [31]. It induces natural immunity and stimulates cellular defence mechanisms through its actions on natural killer cells, cytotoxic T lymphocytes, and modification of the monocyte-macrophage system. It reduces inflammation quickly so that healing can begin [13]. A prospective case series from 2006 reports on a cohort of patients with cystoscopically confirmed late grade 2/3 radiation cystitis following treatment of gynaecologic cancers. 88% of patients showed improvement of haematuria to grade 0/1 after two to four cycles of therapy with WF10 [13].

Another prospective case series of twenty patients with grade three radiation cystitis showed an 80% response rate to TCDO with 30% showing complete response after one cycle [33]. A multicentre randomised control trial showed significantly lower use of antibiotics and antispasmodics in the group treated with WF10. This study also showed a significant reduction in recurrence of haematuria one year following WF10 treatment; however, cystoscopy at one year showed objective improvement in both groups, with no significant difference [12]. Side effects include nausea, headache, and transient anaemia [12, 13]. TCDO/WF10 is not currently licensed for the treatment of radiation cystitis.

#### 6.1.2. Flavoxate Hydrochloride

Flavoxate hydrochloride is an antimuscarinic medication used to treat lower urinary tract symptoms. A nonrandomised, uncontrolled, unblinded study of 34 patients with urgency following pelvic radiotherapy compared two doses of this medication. Patients receiving 1200 mg per day demonstrated improvement on urodynamics in first desire volume, bladder capacity, and pressure at capacity, compared to patients who received 600 mg per day [14]. Flavoxate hydrochloride is licensed for use in cystitis.

#### 6.1.3. Cranberry Capsules/Juice

Cranberry capsules were initially investigated for prevention of UTIs, but a recent Cochrane review does not support the use of cranberry products for UTI prevention. Cranberries are high in antioxidants including flavonoids, anthocyanins, and proanthocyanidins, but bioavailability is very poor. It should also be noted that standardisation of cranberry products is difficult. A randomised, double blinded, placebo-controlled trial from New Zealand examined the use of cranberry capsules in prevention of development of radiation cystitis in men receiving radiotherapy for prostate cancer. All measures of cystitis on a self-reported quality of life index, with the exception of visible haematuria, were lower in the cranberry capsule arm of the study compared to the placebo group. It should be noted however that the hydration regime for patients was changed during the study [15].

Another randomised, double blinded, placebo-controlled trial from Scotland assessed the use of cranberry juice in prevention of radiation cystitis in patients undergoing pelvic radiotherapy for bladder or cervical cancer. They found no significant difference in rates of cystitis between the groups, but the power of the study was significantly undermined by poor compliance, underrecruitment, and high baseline levels of urinary toxicity in the study population [16]. A Canadian randomised control trial compared cranberry juice to apple juice for preventing radiation cystitis in men undergoing radiotherapy for prostate cancer and found no significant difference between the two arms [17].

#### 6.1.4. Conjugated Oestrogen

Conjugated oestrogen is thought to have an effect on the vascular wall, resulting in decreased vascular fragility [31]. Studies in this area are lacking with only one small uncontrolled prospective study from 1990 finding that systemic conjugated oestrogen therapy resolved haematuria in a group of patients with severe radiation and/or cyclophosphamide-induced haemorrhagic cystitis [18]. Conjugated oestrogen is not licenced for use in radiation cystitis.

### 6.2. Intravesical Treatments

Several different intravesical agents have been used in the treatment of radiation cystitis. High quality evidence in support of many of these agents is lacking. Aluminium has appeared historically in the literature in a number of case reports outlining severe refractory haemorrhagic cystitis. It was thought to act as an astringent and cause contraction, wrinkling, and blanching of the surface of the bladder. No studies exist to support its use. Prostaglandins have been used in the gastrointestinal tract to prevent mucosal ulceration and are postulated to work similarly in the bladder, but there are no studies to support this [31]. Outlined below are some of the more commonly used agents and their relevant evidence (Table 4).

#### 6.2.1. Botulinum Toxin A

A retrospective review of six patients with radiation cystitis following pelvic radiotherapy for prostate and cervical cancer suggested some benefit from intravesical botulinum toxin A injection. In five of the six patients, there was an increase in mean bladder capacity and a moderate reduction in urinary frequency from fourteen to eleven episodes per day. No side effects were reported [19]. Botulinum toxin A is currently licenced for the treatment of overactive bladder.

#### 6.2.2. Hyaluronic Acid and Chondroitin Sulfate

Hyaluronic acid is a major mucopolysaccharide that contributes to the protective function of the urothelium. It is also thought to have immunomodulatory properties and to enhance connective tissue healing. Chondroitin sulfate and hyaluronic acid are components of the glycosaminoglycan layer of the bladder. A randomised control trial compared intravesical treatment with hyaluronic acid to hyperbaric oxygen therapy and found that hyaluronic acid provided a significant reduction in pelvic pain and voiding frequency up to eighteen months of follow-up. Hyaluronic acid was equivalent to hyperbaric oxygen therapy for ameliorating haematuria [20].

A nonrandomised prospective cohort study of a mixed group of patients with both chemical and radiation-induced cystitis reported a significant increase in bladder capacity, relief of pelvic pain, and dysuria in 97% of patients with intravesical treatment with hyaluronic acid [34]. A small, nonrandomised case-control study looked at self-reported symptom scores in a group of patients who received intravesical chondroitin sulfate while undergoing pelvic radiotherapy for cervical cancer. Over an eight-week study period, patients who received intravesical chondroitin sulfate scored lower than control patients in the areas of overactive bladder, incontinence, obstructive voiding, and pelvic pain [21]. A small prospective pilot study looked at intravesical treatment with hyaluronic acid and chondroitin sulfate in patients with radiation cystitis following radiotherapy for prostate cancer. A significant reduction in nocturia following treatment was noted; however, the absence of a control group in this trial makes definitive conclusions about treatment efficacy difficult [35]. Hyaluronic acid is licenced for use in interstitial cystitis/painful bladder syndrome.

#### 6.2.3. Formalin

Formalin precipitates cellular proteins in the mucosa of the bladder which occludes telangiectatic tissue and small capillaries. It is extremely caustic to skin and precautions apply when handling this substance [31]. Reports of use of intravesical formalin are relatively small retrospective reviews and rates of resolution of haematuria with intravesical therapy range from 70 to 89%. However, rates of serious complication are as high as 30% [22–24]. Serious complications included bilateral hydronephrosis with anuria, vesicovaginal fistula, and death (*n* = 2/41, due to sepsis) [24]. Five patients in a series of 35 required subsequent urinary diversion due to complications from intravesical formalin therapy [22]. 1% formalin is as effective as higher concentrations and has less associated morbidity [22].

#### 6.2.4. Polydeoxyribonucleotides (PDRN)

Polydeoxyribonucleotides reduce inflammation and improve tissue perfusion and angiogenesis. Evidence for their use in radiation cystitis is sparse. One small prospective observational cohort study of eight patients with late radiation cystitis suggested an improvement in reported cystitis symptoms following biweekly PDRN bladder instillations for two months [25].

#### 6.2.5. Early Placental Extract

Growth factors and angiogenic factors have been identified in early placenta and this has been reported for the healing of venous ulcers [31]. One nonrandomised case-control study reports that patients receiving intravesical placental extract have symptomatic and cystoscopic improvements in radiation cystitis compared to a saline control group over a study period of twelve months [26].

### 6.3. Hyperbaric Oxygen

Hyperbaric oxygen therapy causes hyperoxygenation of tissues, capillary growth into scarred submucosal tissues, and neovascularisation of the bladder. Studies reporting on outcomes of hyperbaric oxygen therapy are mostly retrospective reviews of reasonably large numbers of patients who have failed conservative management of radiation-induced haemorrhagic cystitis. The usual course of treatment involves thirty-five to forty sessions of ninety to one hundred minutes each, five days per week, breathing 100% oxygen at 2-atmosphere absolute pressure per session. Success rates range from 76 to 95% for short-term results and from 72 to 83% for long-term results where success is defined as a symptomatic and/or cystoscopic improvement in radiation cystitis [29, 36–40]. Rates of cure range from 57 to 92%, where cure is defined as complete resolution of visible haematuria [38, 39, 41–44]. Earlier studies with smaller numbers report poor long-term outcomes, with one group reporting 45% of patients who initially responded to hyperbaric oxygen therapy requiring eventual urinary diversion for recurrent haemorrhagic cystitis [45]. However, more recent retrospective studies with larger patient numbers report recurrence rates of 0.12 per year [46]. The longest follow-up was eleven years and this group reported that 74% of patients had no recurrence of symptoms [37]. Outcome also depends on the severity of haematuria prior to treatment and the type of radiotherapy used [36, 37]. Retrospective reviews of large numbers of patients suggest that longer time from onset of haematuria to commencement of hyperbaric oxygen therapy results in significantly poorer clinical response, with optimal results obtained when therapy is commenced within six months of onset of haematuria [36, 37, 47]. The main reported side effect of hyperbaric oxygen therapy is otalgia in 33% of patients [29].

### 6.4. Interventional Procedures

Options for interventional radiological or surgical management of radiation cystitis are limited to case reports or very small case series.

#### 6.4.1. Surgery

Surgical options include cystoscopic management of bleeding, long-term percutaneous nephrostomies, and urinary diversion with or without cystectomy; however, small and large bowel can often be compromised by radiation. A retrospective review of cystoscopic management of radiation cystitis reported that the majority of patients required more than one cystoscopy to attain resolution of haematuria [48]. Cutaneous ureterostomies are reported in the literature as a last resort in intractable radiation-induced haemorrhagic cystitis [49]. A retrospective review of twenty-eight patients with intractable radiation cystitis after radiotherapy for cervical cancer who underwent ileal conduit diversion with or without concomitant vesicovaginostomy suggested that early diversion is prudent in severe disease [50].

#### 6.4.2. Interventional Radiology

Selective radiological embolization of vesical arteries is a minimally invasive option, but no long-term follow-up with large numbers is described.

#### 6.4.3. Argon Beam Coagulator (ABC)

A case series of seven patients with refractory radiation cystitis, who had failed various treatments including intravesical agents and radiological embolization, reports good outcomes with cystoscopic use of an argon beam coagulator. The bladder is distended with carbon dioxide and the ABC is used to treat telangiectatic regions of the bladder. No patients required further catheterisation or blood transfusions. One patient required a hospital readmission for haematuria but this settled spontaneously [51].

#### 6.4.4. Laser

Two small case series of patients with radiation-induced haemorrhagic cystitis report clearance of haematuria after one treatment with the GreenLight Xcelerated Performance System (XPS) or GreenLight potassium-titanyl-phosphate (KTP) laser [52, 53]. A second case series of twenty patients undergoing endoscopic treatment with the GreenLight KTP laser reported a 92.3% rate of resolution of haematuria, although 25% of patients required multiple treatments. The average haematuria-free interval was 11.8 months (1–37 months) [54]. A larger series of forty-two patients reported that thirty-nine patients achieved resolution of haematuria after one session of endoscopic neodymium: YAG laser coagulation under local anaesthesia [55].

## 7. Conclusions

High quality evidence describing the management of radiation cystitis is sparse. Reports of evolving interventional techniques such as laser coagulation of haemorrhagic cystitis continue to emerge; however, radiation cystitis remains a difficult condition to treat. The strongest evidence exists for systemic therapy with TCDO/WF10, cranberry capsules, hyperbaric oxygen therapy, and intravesical hyaluronic acid, but even evidence for these modalities is not of high quality. In the absence of robust evidence for any one treatment modality, most patients are managed supportively in the first instance. Ultimately, most patients require multimodal treatment for curative purposes. In future, large randomised trials exploring emergent management strategies are required in order to strengthen evidence-based treatment strategies.

## Figures and Tables

**Table 1:** The proposed pathophysiology of radiation cystitis [1, 9, 10].

Anatomical location in bladder	The proposed mechanism of radiation damage
Urothelium	Nuclear irregularity
	Cellular oedema
	Increased cytoplasmic elements
	Disruption of tight junctions & polysaccharide layer

Vasculature	Vascular endothelial cell oedema
	Endothelial cell proliferation
	Perivascular fibrosis

Muscle	Smooth muscle oedema
	Replacement of smooth muscle with fibroblasts
	Increased collagen deposition
	Vascular ischaemia of bladder wall

**Table 2:** Radiation Therapy Oncology Group Late Radiation Morbidity Scoring Schema [11].

Grade	Presentation
Grade 0	Normal

Grade 1	Slight epithelial atrophy
	Minor telangiectasia
	Microscopic haematuria

Grade 2	Moderate frequency
	Generalised telangiectasia
	Intermittent macroscopic haematuria

Grade 3	Severe frequency and dysuria
	Severe generalised telangiectasia with petechiae
	Frequent haematuria
	Reduction in bladder capacity (<150 cc)

Grade 4	Necrosis
	Contracted bladder (<100 cc)
	Severe haemorrhagic cystitis

**Table 3:** The proposed systemic treatments for radiation cystitis and their levels of evidence.

Systemic agent	Level of evidence	Data
TCDO/WF10	1b	Reduction in use of antibiotics and antispasmodics
		Reduction in recurrence of haematuria at one year [12]
		Side effects include nausea, headache, and anaemia [13]

Flavoxate hydrochloride	2b	Higher doses of 1200 mg improve urodynamic measures [14]

Cranberry products	1b	Reduction in cystitis symptoms with cranberry capsules [15]
		No evidence for use of cranberry juice [16, 17]

Conjugated oestrogen	3	Resolution of haematuria in severe haemorrhagic cystitis [18]

**Table 4:** The proposed intravesical treatments for radiation cystitis and their levels of evidence.

Intravesical agent	Level of evidence	Data
Botulinum toxin A	4	Increase in bladder capacity
		Reduction in urinary frequency from 14 to 11 episodes
		No side effects reported [19]

Hyaluronic acid	1b	Significant reduction in voiding frequency
		Significant reduction in pelvic pain
		Reduction of haematuria equal to that of hyperbaric oxygen
		Side effects: increased rate of UTIs at 6 months [20]

Chondroitin sulfate	2b	Reduction in self-reported bladder symptoms
		No side effects reported [21]

Formalin	4	Significant reduction in haematuria
		High complication rate including mortality [22–24]

Polydeoxyribonucleotides	3	Improvement in reported cystitis symptoms [25]

Early placental extract	3B	Symptomatic improvement in radiation cystitis
		Cystoscopic improvement in radiation cystitis [26]

**Table 5:** Levels of evidence [27].

Level of evidence	Type of evidence
1a	Systematic review (with homogeneity) of randomised control trials

1b	Individual randomised control trials (with narrow confidence intervals)

1c	All or none randomised control trials

2a	Systematic review (with homogeneity) of cohort studies

2b	Individual cohort study or low quality randomised control trials

2c	Outcomes research

3a	Systematic review (with homogeneity) of case-control studies

3b	Individual case-control study

4	Case series (and poor quality cohort and case-control studies)

5	Expert opinion
